# Comparative analysis and process optimization for manufacturing CAR-T using the PiggyBac system derived from cryopreserved versus fresh PBMCs

**DOI:** 10.1038/s41598-025-89686-7

**Published:** 2025-02-11

**Authors:** Zenghui Xu, Ruyue Wang, Yuanjian Xu, Ruijuan Qiu, Jiangrui Chen, Linfeng Liu, Qijun Qian

**Affiliations:** 1 Shanghai Cell Therapy Group Co., Ltd, 1535 Yuanguo Road, Shanghai, 201805 Shanghai China; 2 Shanghai Cell Therapy Research Institute, 1585 Yuanguo Road, Shanghai, 201805 Shanghai China; 3 Shanghai University Mengchao Cancer Hospital, 118 Qianyang Road, Shanghai, 201805 Shanghai China; 4 School of Medicine, Shanghai University, 99 Shangda Road, Shanghai, 200444 Shanghai China

**Keywords:** CAR-T, Cryopreserved PBMCs, PiggyBac, Electroporation, Process optimization, Cancer, Immunology

## Abstract

Chimeric antigen receptor T (CAR-T) therapy holds promise for cancer treatment but faces challenges with using fresh patient cells, including manufacturing failures and logistical hurdles. Cryopreserved peripheral blood mononuclear cells (PBMCs) offer a potential solution, and while lentiviral processes have been reported for generating CAR-T from these cells, few studies have demonstrated successful PiggyBac electroporation methods. Therefore, the objectives of our study were twofold: Firstly, to conduct a comparative study on cryopreserved PBMCs, fresh PBMCs, and their respective preparations of CAR-T. Secondly, to establish a PiggyBac electroporation CAR-T preparation process using cryopreserved PBMCs through process optimization. The results revealed that long-term frozen PBMCs viability in a relatively stable manner. CAR-T generated from cryopreserved PBMCs exhibited comparable expansion potential, cell phenotype, differentiation profiles, exhaustion markers, and cytotoxicity against human ovarian cancer cell line (SKOV-3) cells to those derived from fresh PBMCs. Moreover, through process optimization, we further enhanced the proliferation and toxicity of CAR-T. This approach has the potential to revolutionize the CAR-T production model by utilizing healthy donor cells instead of patient cells. This shift could mitigate issues affecting treatment efficacy, such as suboptimal cell condition following illness or delays in cell preparation.

## Introduction

The burden of cancer incidence and mortality is increasing rapidly worldwide^1,2^. Conventional standardized treatments have yielded unsatisfactory results for many patients, particularly those in advanced stages. CAR-T therapy is an innovative cancer immunotherapy. It utilizes the patient’s own T cells, which are genetically engineered, expanded in vitro and then infused back into the patient’s body to enable them to recognize and attack cancer cells. CAR-T therapy has shown promising efficacy in the treatment of cancer, especially in hematologic tumors such as acute lymphoblastic leukemia (ALL), chronic lymphocytic leukemia, and lymphoma^3–5^. Although the therapeutic effect of CAR-T on solid tumors is not as remarkable as that on hematological tumors, it still holds considerable development prospects^3,6^.

In general, CAR-T are derived from T cells isolated from the patient’s PBMCs or leukapheresis. The CAR gene is introduced into T cells through viral or non-viral systems^7,8^. The efficacy of CAR-T therapy is largely dependent on the quality of the T cells, and therefore, the quality of the patient’s immune cells is closely related to the efficacy of the therapy^4,9^. A number of studies have shown that the composition of patient’s immune cells differs significantly from that of healthy people^10–12^. Healthy donors may have a greater proportion of CD4^+^T cells than patients with ALL, and have a greater capacity for expansion and a greater variety of TCRs during CAR-T culture^13,14^. The phenotype of T cells differentiation plays an important role in the ongoing immune response^15,16^. Currently, many CAR-T are manufactured to preserve the stem-like status of the CAR-T by shortening the culture time, thus improving the product’s efficacy^17–19^. Studies have shown that T cells isolated from healthy individuals are less differentiated and retain the stem-like status of the cells better in culture^18,20,21^. Prior chemotherapy experienced by a patient may impact the quality and function of T cells^22^, reducing the function of CAR-T product^15,23,24^. In order to preserve the characteristics of T cells well and retain the therapeutic effect of CAR-T, extracting PBMCs at a healthy stage and storing them by freezing for later use would be an effective way.

Despite the relative effectiveness of viral transduction and the successful demonstration of CAR-T preparation from frozen PBMCs, the production of CAR-T products still presents challenges, viral preparation leads to high costs and cargo capacity is limited to 7–10 kb^25^, preventing the delivery of larger genes. Immunogenicity has also been reported when it is directly administered^26–29^. The PiggyBac transposon overcomes some of these limitations. For example, plasmids are easier to prepare and do not require viruses, which greatly reduces the cost of CAR-T preparation^30^. Additionally, with a cargo capacity up to 100 kb, it increases the ability to transduce larger DNA fragments. It has been shown that transposons can significantly reduce the incidence of immunogenicity^25,31^. However, there are few reports on the use of non-viral transfection for the preparation of CAR-T from frozen PBMCs. It is not yet clear whether there are differences in CAR-T quality attributes between those prepared from frozen PBMCs and fresh PBMCs utilizing non-viral electroporation. Therefore, the aim of our study is to conduct a comparative analysis of cryopreserved PBMCs, fresh PBMCs, and their respective preparations of CAR-T, and to explore the feasibility of generating CAR-T from cryopreserved PBMCs using the PiggyBac transposon electroporation system.

## Results

### Impact of cryopreservation duration on viability and phenotypic stability in PBMCs

**Fig. 1:**
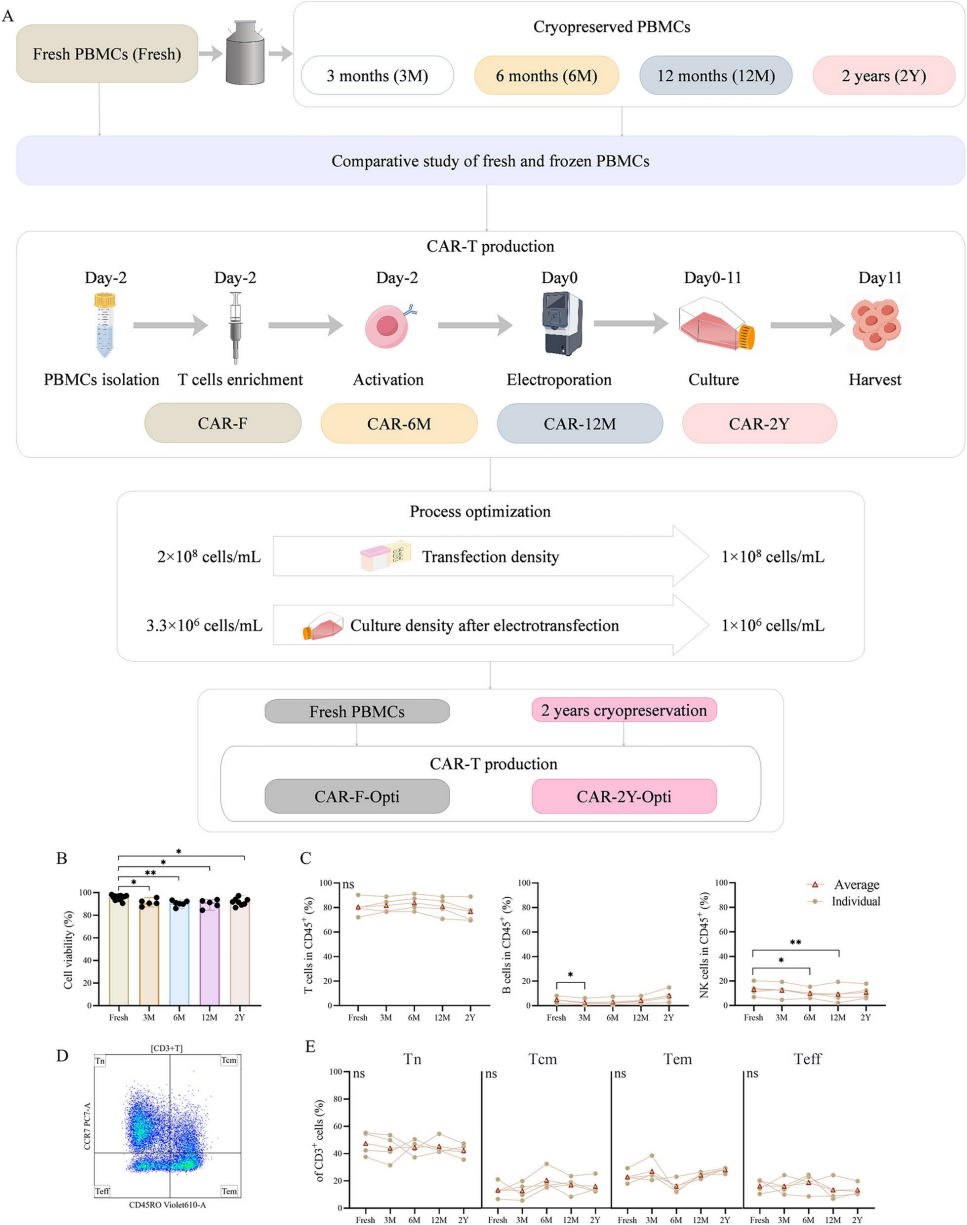
Comparison of cell viability and phenotype of cryopreserved versus fresh PBMCs. (**A**) Experimental workflow. (**B**) Viability of PBMCs (n = 5–14). (**C**) Phenotypic analysis (n = 4). (**D**) The logic diagram for flow cytometry analysis. (**E**) Differentiation state of T cells (n = 4). (**B**) Median with 95% Confidence Interval (CI). (**C**), (**E**) Individual data and average. Statistical analysis was conducted using the Kruskal–Wallis test (**B**) and the Friedman test (**C, E**), with a *p* < 0.05 considered statistically significant. * *p* < 0.05, ** p < 0.01, “ns” indicates no significant difference.

To analyze the effect of freezing on the function of PBMCs, we compared PBMCs with different freezing times with fresh PBMCs and prepared CAR-T cells separately to study the effect of frozen PBMCs on the function of CAR-T cells. The specific process is shown in Fig. 1A**.** The viability and phenotype of PBMCs, particularly the T cells phenotype, are crucial for the success of CAR-T manufacturing. We investigated the effects of different cryopreservation durations—3 months (3 M), 6 months (6 M), 12 months (12 M), and 2 years (2Y)—on PBMCs viability. The study revealed that, although there was a significant difference in cell viability between fresh PBMCs and those after cryopreservation, the actual decrease was only 4.00% to 5.67% (Fig. 1B). Moreover, we measured the viability of PBMCs frozen for 3.5 years, with an average viability of 90.95% (**Supplementary Fig. 1**). The viability did not change with the extended freezing time, suggesting that long-term cryopreservation can effectively preserve PBMCs viability remained relatively constant. Furthermore, multicolor flow cytometry analysis revealed a decrease in the proportions of natural killer (NK) cells and B cells following cryopreservation, presumably attributable to their sensitivity to hypothermic conditions. Notably, however, the proportion of T cells remained relatively stable (Fig. 1C), indicating that CAR-T preparation was unaffected, as it primarily derives from CD3^+^T cells. Previous studies have shown that Tnaïve and central memory T cells (Tcm) enhance CAR-T activation, persistence, and effector function, improving therapeutic efficacy^21,24,32^. T cells differentiation states were investigated by staining the cells with fluorochrome-labeled antibodies specific to CD45RO and CCR7 (Fig. 1D). This analysis indicated no significant changes in Tn (CD45RO^-^CCR7^+^) and Tcm (CD45RO^+^CCR7^+^) proportions in T cells post-cryopreservation compared to fresh samples (Fig. 1E). These results collectively endorse cryopreserved PBMCs as a feasible alternative for CAR-T preparation.

### Long-term cryopreservation of PBMCs did not affect the function of mesoCAR-T

**Fig. 2:**
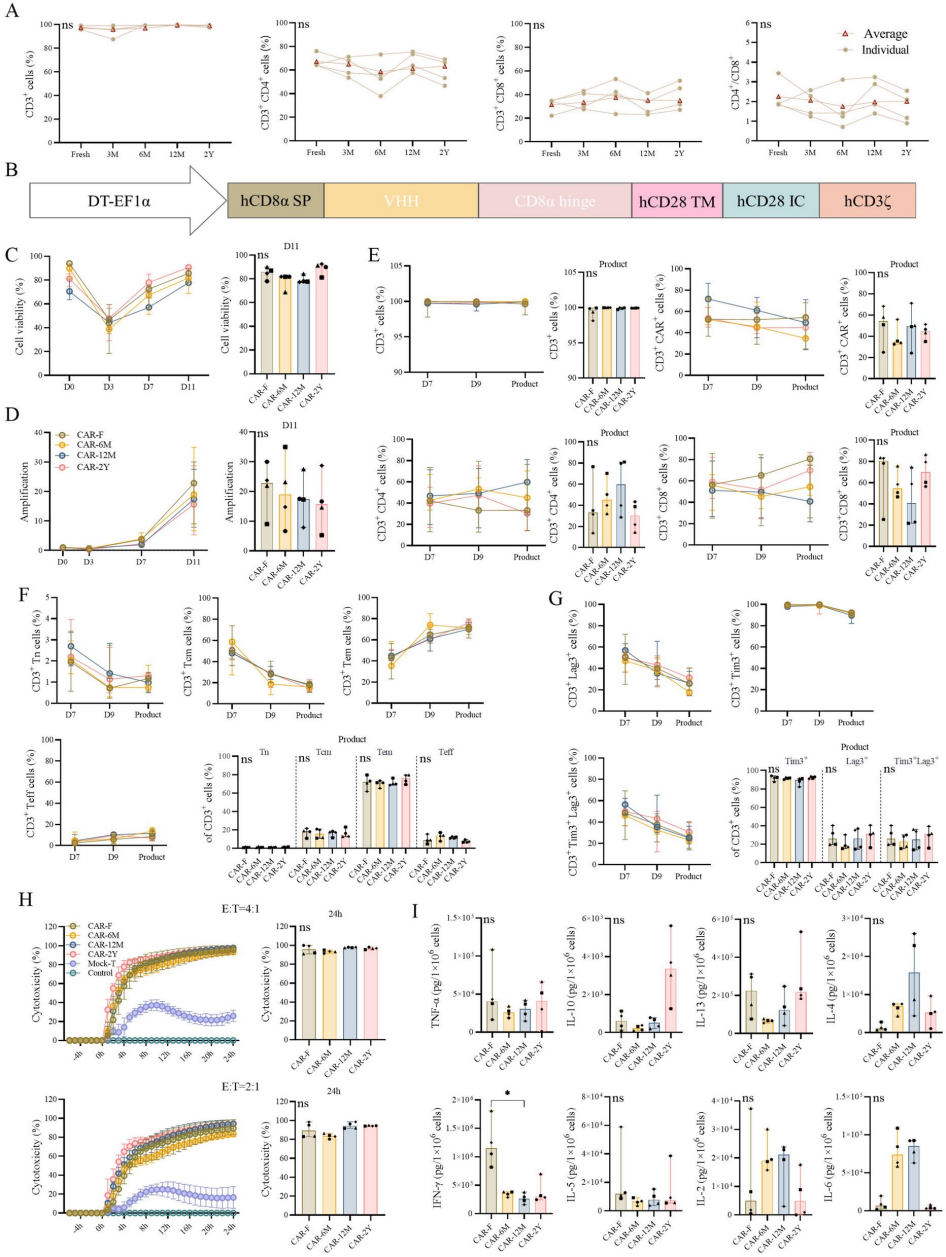
Comparison of quality attributes of mesoCAR-T derived from cryopreserved and fresh PBMCs. (**A**) Proportions of CD3^+^, CD3^+^CD4^+^, and CD3^+^CD8^+^ cells post magnetic bead enrichment (n = 4). (**B**) Structure of the mesoCAR. Viability (**C**) and cell expansion potential (**D**) during mesoCAR-T manufacturing (n = 4). The histogram in (**C**) and (**D**) represent the viability and amplification of CAR-T cells on the 11th day of culture, respectively. (**E**) Percentage of CD3^+^, CD4^+^, CD8^+^ and mesoCAR^+^ cells (n = 4). Comparative analysis of the differentiation states (**F**) and exhaustion (**G**) (n = 4). The histogram in E–G present the statistical analysis of the CAR-T product. (**H**) Toxicity capacity curves, tested at various E:T (4:1 or 2:1) against SKOV-3 cells within 24 h (n = 4), and the toxicity at 24 h was analyzed statistically. (**I**) Cytokine secretion (n = 4). The data presented in (**A**) includes both individual and average values, whereas (**C-I**) show median with 95% CI. Dots of the same shape represent data from one individual. The Kruskal–Wallis test (**A**) and the Friedman test (**C-I**) were used for statistical analysis, with a *p* < 0.05 was considered significant. *p < 0.05, “ns” indicates no significant difference.

To conduct a more in-depth investigation into the potential influence of utilizing cryopreserved PBMCs for CAR-T manufacturing on the phenotypic characteristics and functional capabilities, we conducted a 2 years study using samples from four healthy donors **(**Fig. 1A). Following CD4/CD8 magnetic beads enrichment from cryopreserved PBMCs, we assessed CD3^+^ purity and CD3^+^CD4^+^, and CD3^+^CD8^+^ proportions, which were unaffected by long-term cryopreservation **(**Fig. 2A). After 48 h of activation, the electroporation of the mesothelin (MSLN) CAR vector (Fig. 2B) was performed, followed by cell culture for 11 days to generate mesoCAR-T. Our findings showed no significant difference in cell viability between mesoCAR-T derived from fresh and cryopreserved PBMCs (Fig. 2C). While previous research indicated cryopreservation could affect cell proliferation^33,34^, our results showed only slight reductions in proliferation across the 6 M, 12 M, and 2Y timelines, with no significant impact (Fig. 2D). Phenotype assessments throughout the culture process revealed consistent mean CD3^+^ purity, CD4^+^, CD8^+^ cells, and transfection efficiency across both fresh and cryopreserved PBMCs-derived mesoCAR-T (Fig. 2E). Tn and Tcm cells, although gradually decreasing with culture time, showed no significant differences between fresh and cryopreserved samples at harvest (Fig. 2F). This consistency was also reflected in T cells exhaustion markers (Fig. 2G), suggesting cryopreservation does not compromise mesoCAR-T persistence.

To verify the potential impact on mesoCAR-T functionality, we conducted real time cellular analysis (RTCA) cytotoxicity and cytokine release assessments on mesoCAR-T derived from both fresh PBMCs (CAR-F) and cryopreserved PBMCs stored for 6 months (CAR-6 M), 12 months (CAR-12 M), 2 years (CAR-2Y). The non-mesoCAR-expressing T cells group (Mock-T), and the target cell only group (Control) served as control groups. At an effector-to-target ratio (E:T) of 4:1, cytotoxicity in both CAR-F and CAR-2Y was comparable: 91.02%−100.00% and 95.46%−98.07%, respectively (Fig. 2H). Similarly, when using a 2:1 ratio, no statistically significant difference in cytotoxicity was observed (Fig. 2H). We conducted a statistical analysis of cytokine secretion among different groups, and the results showed that IFN-γ has a significant decrease in CAR-12 M compared to CAR-F **(**Fig. 2I**)**. Although studies have reported that IFN-γ is related to cell cytotoxicity^5^, our results indicate that the cytotoxic function of mesoCAR-T remains unaffected **(**Fig. 2H**)**. No systematic changes were observed in the secretion of other cytokines (IL-6, IL-10, IL-5, IL-4, IL-13, IL-2, TNF-α) due to the cryopreservation of PBMCs (Fig. 2I). These results indicate that there are similar functional profiles for mesoCAR-T from both fresh and cryopreserved PBMCs.

### Process, not freezing, is key to mesoCAR-T preparation in PiggyBac transposon electroporation

**Fig. 3:**
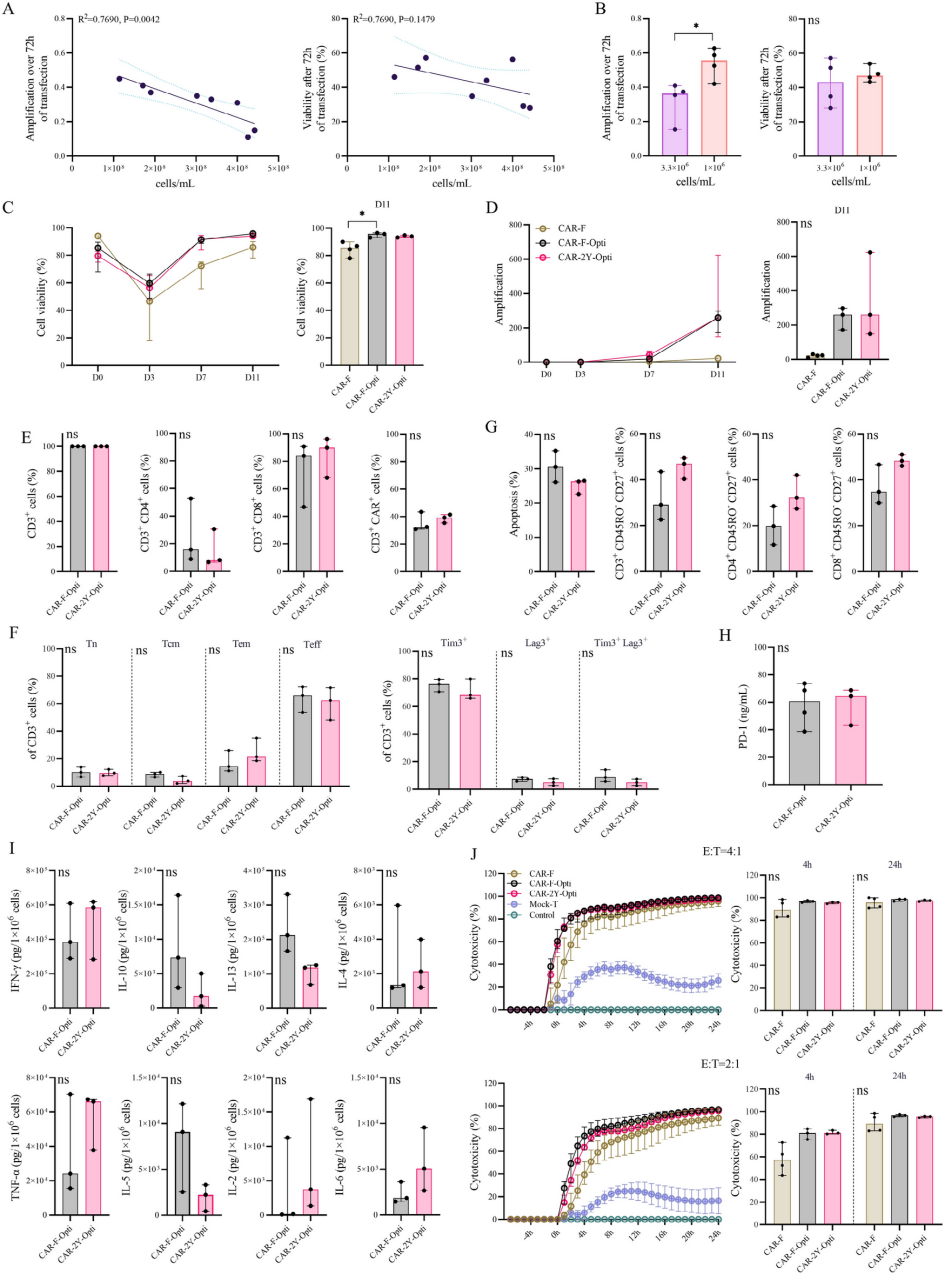
Optimized process for enhanced expansion and functionality of mesoCAR-T from cryopreserved PBMCs. (**A**) Optimization of electroporation density. (**B**) Optimization of culture density after electroporation. Viability (**C**) and expansion potential (**D**) during mesoCAR-T manufacturing. (**E**) Percentages of CD3^+^, CD4^+^, CD8^+^, and mesoCAR^+^ cells (n = 3). (**F**) Differentiation and exhaustion states (n = 3). (**G**) Proportions of apoptotic cells and CD3^+^, CD4^+^, CD8^+^ subsets expressing CD45RO^-^CD27^+^ (n = 3). (**H**) The secretion level of PD-1 at an E:T = 2:1 (n = 3-4). (**I**) Cytokine secretion (n = 3). (**J**) The cytotoxic capacity curves at different E:T ratios (4:1 or 2:1) against SKOV-3 cells, and the cytotoxicity at 4 h and 24 h is shown in the histogram. Data is presented as median with 95% CI. Each dot represents an individual. Statistical analysis was conducted using the linear regression (**A**), Kruskal–Wallis test (**C, D, J**) or Mann Whitney test (**B, E, F, G, H, I**), with a *p* value < 0.05 indicating significance. * *p* < 0.05, “ns” indicates no significant difference.

Although mesoCAR-T were successfully generated from PBMCs frozen for 2 years, there was a significant decline in cell viability on day 3 post-electroporation, with amplification reaching only 21.18-fold, which was notably lower than that achieved via viral transduction^35,36^. This prolongation of culture may lead to increased mesoCAR-T exhaustion^18,19^, a significant drawback in electro-transfection usage. To address this issue, we evaluated the effects of different electroporation densities (1 × 10^8^—5 × 10^8^cells/mL) and different post-electroporation culture densities (3.3 × 10^6^ cells/mL and 1 × 10^6^ cells/mL) on cell expansion and viability. The results showed that high-density electroporation slightly affected cell viability (*p* = 0.1479); however, it reduced amplification over 72 h (*p* = 0.0042) (Fig. 3A). Furthermore, adjusting culture density from 3.3 × 10^6^ to 1 × 10^6^ cells/mL resulted in an average amplification increase of 67.23% within 72 h without compromising viability (Fig. 3B), significantly enhancing frozen PBMCs preparation for mesoCAR-T electro-transfection. In summary, we believe that the electroporation density of 1 × 10^8^ cells/mL and the post-electroporation culture density of 1 × 10^6^ cells/mL can achieve better mesoCAR-T expansion, and thus we regarded this as the optimized process.

To determine the potential impact on mesoCAR-T functionality due to process optimization, we conducted mesoCAR-T production using fresh PBMCs (CAR-F-Opti) and PBMCs cryopreserved for 2 years (CAR-2Y-Opti), compared with CAR-F (electroporation density of 2 × 10^8^ cells/mL, post-electroporation culture density of 3.3 × 10^6^ cells/mL). The results indicated that optimized electroporation increased amplification efficiency by 109.12% and 210.88% over 72 h for both fresh and frozen PBMCs (not shown). Under the optimized conditions, CAR-F-Opti and CAR-2Y-Opti viability increased by 12.05% and 10.76% (Fig. 3C), and amplification increased by 186.56% (*p* = 0.1098) and 304.55% (*p* = 0.0764) over the entire preparation cycle on average (Fig. 3D). Unfortunately, there was no significant difference, which may be due to insufficient sample size and inter-donor variability within groups. CD3^+^ purity, proportion of CD3^+^CD4^+^ and CD3^+^CD8^+^, and the positivity rates of mesoCAR-T were unaffected by freezing (Fig. 3E). These assessments revealed no reduction in mesoCAR-T efficacy post-optimization or post-freezing, with phenotype stability confirmed by evaluating T cells apoptosis, differentiation, exhaustion, and CD45RO^-^CD27^+^ markers (Fig. 3G).

To evaluate the cytotoxic activity of mesoCAR-T, we investigated the secretion of cellular cytokines, and conducted simulations of the toxic effects of mesoCAR-T on SKOV-3 cells by referring to the method reported by Zhang^37^, since SKOV-3 is a highly MSLN antigen-expressing cell line. The Mock-T and the target cell only group (Control) were used as control. Programmed death receptor 1 (PD-1) is a crucial immune checkpoint protein that regulates immune system activity. Tumor cells may evade immune response by expressing PD-L1, the ligand of PD-1, to inhibit T cells activity. Therefore, detecting PD-1 secretion is essential for assessing the anti-tumor potency of CAR-T cells. We analyzed the PD-1 secretion of mesoCAR-T obtained from frozen PBMCs and found no significant difference compared to the fresh group (Fig. 3H). Cytokine Release Syndrome (CRS) is one of the common and severe side effects in CAR-T therapy. It is a systemic inflammatory response caused by the release of a large number of cytokines. Studies have shown that the signs and symptoms of CRS are positively correlated with serum levels of IFN-γ, TNF-α, IL-6, and IL-2^38,39^. Other studies have shown that elevated levels of IL-5 and IFN-γ are independent risk factors for immune-related adverse events^40^. In this study, there was no difference in the secretion of the cytokines IFN-γ, IL-6, IL-2, and IL-5 when CAR-T cells were prepared from PBMCs frozen for 2 years compared to the fresh group. IL-10 can mitigate the severity of CRS by inhibiting the production of inflammatory cytokines, such as IL-6 and TNF-α^41^. However, our findings indicated that IL-10 secretion remains unaffected by freezing (Fig. 3I). IL-4 typically activates the immune system to fight cancer^42^. However, an excess of IL-4 protein can lead to CAR-T cell overload and induce exhaustion^43^. IL-13 interacts with its high-affinity receptor IL-13Rα2, inhibiting tumor cell apoptosis and resulting in poor tumor prognosis^44^. We measured the secretion levels of IL-4 and IL-13, and the results showed no significant difference between mesoCAR-T prepared from fresh PBMCs and frozen PBMCs. Under optimized conditions, we observed a rapid tumor response at E:T ratio of 4:1, achieving an 80.00% kill efficiency within 4 h and this efficacy continued to increase over a 24 h period (Fig. 3J). In summary, our optimized regimen enhances both the expansion and efficacy of mesoCAR-T while ensuring safety. These advancements contribute valuable insights towards achieving more reliable treatment outcomes.

## Discussion

Clinical evidence demonstrates the immense potential of CAR-T therapy in treating hematologic malignancies and solid tumors. Efficiently preparing high-quality CAR-T is a pressing concern in optimizing CAR-T therapy. Currently, the two principal methods utilized for the generation of CAR-T are viral transduction and bulk electroporation. Viral transduction, considered the most established method, features stable expression, high transduction efficiency, and perpetual integration into the host cell’s genome^45,46^. However, the manufacturing process for viral vectors is also complex, time-consuming, and expensive^47^, especially for academic centers not equipped with virus production facilities. Resolving the cost issue related to CAR plasmid transfection could significantly reduce the price of CAR-T therapies. Electroporation, by contrast, has ameliorated these issues. This method allows CAR genes to enter T cells through the cell membrane at a low cost and low immunogenicity^48,49^. Consequently, non-viral vectors may emerge as a more universal, flexible, and sustainable solution for future T cells engineering^50^. CAR-T cell preparation utilizes fresh PBMCs sourced primarily from patients battling late-stage cancer. Various physical conditions could pose challenges in harvesting adequate PBMCs, potentially resulting in unsatisfactory efficacy of the produced CAR-T. Unfortunately, there were high rates of patient death while awaiting CAR-T production^22^. Cryopreserving PBMCs could offset these problems. Existing literature documents preparation of CAR-T from cryopreserved PBMCs using viral transduction^22,36,51^, however, few published reports have yet provided a detailed account of doing so with electroporation.

In our study, we proposed a process to produce mesoCAR-T by electroporation of cryopreserved PBMCs and compared the functionality with that of mesoCAR-T prepared from fresh PBMCs. A slight decrease in PBMCs survival was observed after cryopreservation (4.00%−5.67%), but the viability of PBMCs frozen for 3 months to 3.5 years did not decrease with extended freezing time. This may be due to the fact that cryopreservation damage is primarily caused by ice crystal damage during the cooling and thawing processes^52,53^, and cryopreservation time is not a key factor affecting cell quality. There are reports indicating that cells frozen for 30 months can successfully prepare CD19 CAR-T through viral transduction^36^, and umbilical cord blood frozen for 10 years can successfully perform NK expansion^54^, which is consistent with our results. Importantly, since CD3^+^ T cells are the primary source of CAR-T, high viability of PBMCs and a stable proportion of CD3^+^ T cells ensure that downstream CAR-T production remains viable.

Moreover, we examined the phenotypes of mesoCAR-T manufactured and discovered that their purity, viability and mesoCAR^+^characteristics exhibited similarities. It has been reported in the literature that freezing affects cell expansion^34. ^In our study, although the amplification of mesoCAR-T derived from cryopreserved PBMCs was slightly reduced after cryopreservation, it was not significantly different from the fresh group. This finding indicates the feasibility of preparing mesoCAR-T through the electroporation of cryopreserved PBMCs. Subsequently, we evaluated the capacity of mesoCAR-T to eliminate tumor cells. Consistent with previous reports^55^, we observed cytokine secretion decrease in the group with cryopreserved PBMCs, especially in IFN-γ levels. Importantly, this decrease did not affect the anti-tumor effectiveness of mesoCAR-T. Summarizing, this study demonstrates a successful method for preparing clinically valuable mesoCAR-T from cryopreserved PBMCs using electroporation.

However, even though we successfully prepared mesoCAR-T from long-frozen PBMCs, the amplification as well as the viability of the mesoCAR-T, especially on the third day after transfection, was significantly reduced. Therefore, we performed the optimization of the transfection process. The optimized process proved to be crucial in minimizing the adverse effects of cryopreservation. By improving expansion efficiency and maintaining cellular function, the optimized process narrowed the gap between using fresh and cryopreserved PBMCs, especially for amplification and IFN-γ secretion. And the optimized process resulted in mesoCAR-T responding faster to tumor cells and thus killing the cells, highlighting the fact that process improvements are key to successful mesoCAR-T manufacturing. This coincides with previous findings that emphasize the importance of manufacturing protocols in preserving T cells function^17^. In a word, after 11 days of in vitro culture, 1 × 10^7^ frozen PBMCs per yielded at least 1.48 × 10^9^ mesoCAR-T cells, which contain more than 30% CAR^+^ cells. Moreover, this process manufacturing platform enables direct cGMP certification.

The unpredictability nature of the cancer frequently necessitates patients to undergo PBMCs enrichment for CAR-T preparation post-diagnosis or even during the advanced stages. This timing could potentially diminish the efficacy of CAR-T therapy, as the extraction process can be hindered by the patient’s existing medical condition^56,57^. So we are proposing the cryopreservation of PBMCs. Healthy individuals can opt for PBMCs cryopreservation at any convenient time, which is not very costly as only about 50–200 mL of peripheral blood is taken and PBMCs are isolated and stored in liquid nitrogen. As soon as CAR-T are required for treatment, we can instantly start with CAR-T preparation without concern for the patient’s existing medical condition. This enables patients to receive timely CAR-T treatment without any delay.

In our study, we successfully prepared mesoCAR-T from cryopreserved PBMCs via electroporation and did not find that cryopreservation led to a reduction in cytotoxicity. We expect that further optimization of our method will lead to an efficient, low-cost, and functionally stable CAR-T preparation process. Of course, there are some limitations to our current study. Firstly, our testing was limited to samples frozen for 2 years. Although there are reports in the literature that cells frozen for longer periods (up to 10 years) can successfully achieve NK expansion, further research is needed to investigate the effects of longer freezing durations on the preparation of CAR-T using non-viral systems. Secondly, when evaluating cryopreserved PBMCs using optimized processes, donor matching between fresh and frozen samples was not achieved, which may introduce experimental errors attributed to inter-donor variability. Additionally, although we successfully prepared mesoCAR-T using different plasmid systems **(Supplementary Table 1),** there is a lack of validation for other target-specific CAR-T products. Thirdly, our study primarily focused on the *in-vitro* functionality of CAR-T prepared from cryopreserved PBMCs from healthy donors, and clinical data support is still lacking. And there are fewer clinical cases on PiggyBac CAR-T to determine the likelihood of transposon-induced tumorigenesis. To address these limitations, we have commenced testing with PBMCs frozen for longer durations and plan to conduct animal testing and clinical assays in the future. This will enable us to acquire valuable data for further analysis and to validate the feasibility of our approach in clinical settings.

## Conclusions

In conclusion, our study has successfully demonstrated the generation of high-quality CAR-T from cryopreserved PBMCs utilizing an electroporation-based approach. By comparing our findings with previously published reports and ongoing clinical trials that employ cryopreserved CAR-T, our findings significantly bolster the growing body of evidence that supports utilizing cryopreserved PBMCs for CAR-T production through electroporation. This study not only validates the feasibility of our approach but also has the potential to establish a new benchmark for future CAR-T production, thereby enhancing the accessibility and availability of these therapies to a wider patient population.

## Materials and methods

### Apheresis of peripheral blood mononuclear cells

Leukapheresis of 50–60 mL was obtained from healthy donors using Spectra Optia (Terumo BCT, Lakewood, Colorado), and PBMCs were separated via Ficoll (Cytiva, Uppsala, Sweden) density gradient centrifugation. The collected PBMCs were divided into aliquots of equal number. One portion was immediately processed into mesoCAR-T while the remaining portions were added to CryoStor CS10 (Stemcell, Vancouver, Canada) at 1 ~ 2 × 10^7^ cells/mL and stored in liquid nitrogen for future manufacture of mesoCAR-T at different cryopreservation times. Cell number was quantified using a Cellometer K2 (Nexcelom, Boston, Massachusetts), and cell viability was determined by AO/PI Staining Solution (Nexcelom, Boston, Massachusetts). The study was approved by the Ethics Committee of the Shanghai Liquan Hospital, China (ethics committee number: 20212002-C221). It was performed in compliance with the Declaration of Helsinki. Written informed consent was obtained from all individual participants included in the study.

### Thaw of cells

PBMCs or mesoCAR-T were immediately thawed in a 37 ℃ water bath after being removed from liquid nitrogen. When the cells were just about melted, they were transferred to a PBS/EDTA buffer (Miltenyi Biotech, Begich Gladbach, Germany) containing 0.5% HSA (Hulan Bio, Henan, China). This buffer was double the volume of the cells. The cells were then readied for subsequent experiments.

### Manufacture of mesoCAR-T

CD3^+^ T cells were enriched using CD4 and CD8 MicroBeads (Miltenyi Biotec, Bergisch Gladbach, Germany). Following enrichment, the cells were transferred to AIM-V medium (Gibco, San Francisco, California), supplemented with 25 ng/mL IL-7 (R&D system, Minneapolis, Minnesota), 25 ng/mL IL-15 (R&D system, Minneapolis, Minnesota), and a 5% serum replacement (ThermoFisher, Waltham, Massachusetts). And then added 10μL/mL T Cell TransAct™ (Miltenyi Biotec, Bergisch Gladbach, Germany) to activate T cells at 1 × 10^6^ cells/mL. 48 h later, activated T cells were centrifuged and re-suspended in PiggyBac enzyme mRNA (320 μg/mL), PD-1 plasmid (90 μg/mL) and MSLN plasmid (84 μg/mL) mixture. The mixture was transferred to electroporation cuvettes (Maxcyte, Gaithersburg, Maryland) and subsequently electroporated using the Scalable Transfection System (Maxcyte, Gaithersburg, Maryland), specifically under the Expand T cell-4 program. Following electroporation, cells were incubated at 37 °C for 15–20 min. The cells were then relocated to T75/T25 culture flasks containing complete medium at 37℃. The complete medium is supplemented every 2–3 days to optimize growth conditions. On the 11th day, the cells were washed with physiological saline, added to the cryopreservation solution and stored in liquid nitrogen.

### Culture of tumor cells

SKOV-3 (RRID: CVCL_0532) was obtained from American Type Culture Collection (ATCC, Manzas, Virginia) and stored in liquid nitrogen. They were rapidly resuscitated in a 37 °C water bath before use and cultured in DMEM medium (Corning, New York, America) containing 9% FBS (Gibco, Auckland, New Zealand).

### Flow cytometry analysis

A multicolor flow cytometry was performed to identify the phenotypes of both PBMCs and mesoCAR-T. The phenotypic markers for T cells, NK cells, and B cells were analyzed using specific anti-human antibodies: CD45, CD16, CD3, CD19 and CD56. The differentiation of T cells was marked with CD3, CD45RO and CCR7 antibodies. The purity of the enriched T cells was ascertained via the application of anti-human CD3, CD4 and CD8 antibodies. Moreover, the profiles of mesoCAR-T were identified using CD3, CD4, CD8 and Biotin Conjugated protein from MSLN, Streptavidin-PE antibodies. The differentiation of mesoCAR-T was examined using anti-human CD45RO, CCR7 and CD27 antibodies. Assessment of mesoCAR-T exhaustion was achieved with anti-human Lag-3 and Tim-3 antibodies. The antibodies used for the assay are presented in Table 1. The targeted cells underwent a process of washing in phosphate-buffered saline (PBS) (Solarbio, Beijing, China), incubation with their respective antibody for 15 min at 4℃, followed by a second wash with 4 mL of PBS before being resuspended in 300 μL of PBS. Fluorescence was measured using a CytoFLEX instrument (Beckman, Pasadena, California) and subsequent data were analyzed with Kaluza Analysis software.

**Table 1:** The antibodies employed for staining and flow cytometric analysis of cell phenotypes.

Antibody name	Manufacturer	Catalog Number
Alexa Fluor® 488 anti-human CD3	BioLegend Inc., San Diego, California	300,415
PE anti-human CD16	Thermo Fisher Scientific, Waltham, Massachusetts	12–0168-42
PerCP/Cyanine5 anti-human CD45	BioLegend Inc., San Diego, California	304,028
APC anti-human CD19	BioLegend Inc., San Diego, California	302,212
Brilliant Violet 421 anti-human CD56	BioLegend Inc., San Diego, California	318,318
Alexa Fluor 700 anti-human CD3	BioLegend Inc., San Diego, California	557,943
Alexa Fluor®488 anti-human CD4	BioLegend Inc., San Diego, California	344,618
PerCP-Cyanine5. 5 anti-human CD8a	Thermo Fisher Scientific, Waltham, Massachusetts	45–0088-42
Brilliant Violet 605 anti-human CD45RO	BioLegend Inc., San Diego, California	304,238
PE/Cyanine7 anti-human CCR7	BioLegend Inc., San Diego, California	353,226
Biotin Human Mesothelin protein	Genscript Biotech, Nanjing, China	N/A
PE Streptavidin	BD Biosciences, San Jose, California	554,061
PE/Cyanine7 anti-human Lag-3	BioLegend Inc., San Diego, California	369,310
Brilliant Violet 650 anti-human Tim-3	BD Biosciences, San Jose, California	565,564
BV786 Mouse Anti-Human CD27(L128)	BD Biosciences, San Jose, California	563,327

### Apoptosis detection assay

mesoCAR-T were collected by centrifugation at 400 g for 5 min and washed with PBS, followed by detection of cell apoptosis using the Annexin V-FITC apoptosis detection kit (Beyotime, Shanghai, China). 195 μL Annexin V-FITC binding solution was added to resuspend cells gently. Then 0.5 μL Annexin V-FITC and 10 μL propidium iodide (PI) staining solution were added, followed by gentle mixing, incubation at room temperature (20–25 °C) in the dark for 20 min and subsequently placed it in an ice bath. During the incubation process, cells were resuspended 2–3 times. Flow cytometry analysis was conducted within 1 h. Fluorescence was measured using a CytoFLEX instrument from Beckman, and subsequent data were analyzed with Kaluza Analysis software.

### Cytotoxicity analysis

Real time cellular analysis was used to detect cytotoxicity of mesoCAR-T. First, 1 × 10^4^ SKOV-3 cells per well were incubated in an E-plate 96 (Agilent Technologies, Santa Clara, California). Subsequently, the plate was placed into the xCELLigence RTCA SP instrument (Agilent Technologies, Santa Clara, California) to monitor the growth of tumor cells. The following steps were conducted once the cell index exceeded 1. Within 16–24 h after mesoCAR-T were thawed, we considered two different E:T ratio: 4:1 and 2:1. The corresponding number of positive cells were then seeded into the E-Plate 96 previously seeded with tumor cells. Subsequently, the plate was placed into the xCELLigence RTCA SP instrument to monitor the cytotoxicity effect. After 24 h, the cytotoxicity data were analyzed via xCELLigence RTCA Pro software.

### Cytokines release analysis

For the cytokines release assay, 1 × 10^4^ target cells were co-cultured with mesoCAR-T at an E:T ratio of 4:1 for 24 h. The detection of cytokines IL-5, IL-13, IL-2, IL-6, IL-10, IFN-γ, TNF-α, and IL-4 was performed using the LEGENDplex™ Multi-Analyte Flow Assay Kit (BioLegend, San Diego, California).

### PD-1 secretion assay

For the PD-1 secretion release assay, 1 × 10^4^ target cells were co-cultured with mesoCAR-T at an E:T ratio of 2:1 for 24 h. Post-incubation, the supernatant was stored at −20 °C for enzyme-linked immunosorbent assay.

Human PD-1/PDCD1 protein (ACRO, Beijing, China) was added to enzyme-labeled plates at 100 μg/mL and incubated at 2–8 °C for 16–24 h. Plates were washed three times with wash buffer (PBS containing 0.05% Tween-20). Each well was blocked with PBS containing 1% BSA (Kingmorn, Shanghai, China) at 37 °C for 2 h. Samples were then added and incubated at room temperature for 1 h, followed by washing. Then, 100 μL of 0.025 μg/mL MonoRabTM Rabbit Anti-Camelid VHH Antibody (83E7)—HRP, mAb (GenScript, Nanjing, China) was added and incubated for 0.5 h. After washing, 100 μL/well TMB substrate (Abcam, England, Cambridge) was added and incubated for 10 min, followed by 100 μL stop solution (Abcam, England, Cambridge). Shake gently and mix well, terminate for 10 min and use Spark multimode microplate reader (TECAN, Switzerland, Austrian) for detection.

### Statistical analysis

Analyses were performed using GraphPad Prism 9. Friedman tests were employed for analyzing multiple related groups. Kruskal–Wallis tests were performed on multiple unrelated groups. Unpaired two-tailed Mann–Whitney tests were employed to analyze two unrelated groups. Statistical significance was indicated by *p* values: *p* < 0.05, * p < 0.05, ** p < 0.01, “ns” indicates no significant difference.

## Supplementary Information

Supplementary Information.

## Data Availability

Data are provided within the manuscript or supplemental materials; if any details are needed, please contact the corresponding author on reasonable request.
