# Supplementary material for: Comparative analysis and process optimization for manufacturing CAR-T using the PiggyBac system derived from cryopreserved versus fresh PBMCs
Source: Sci Rep. 2025 Feb 11;15:5023. doi: 10.1038/s41598-025-89686-7 (PMC11814250; doi:10.1038/s41598-025-89686-7)
Supplement: Supplementary file 1 — Supplementary Information. [file 41598_2025_89686_MOESM1_ESM.docx]

**Comparative Analysis and Process Optimization for** **Manufacturing CAR-T Using the PiggyBac System Derived from Cryopreserved versus Fresh PBMCs**

**Zenghui Xu^1, 2, 3*+^· Ruyue Wang^1+^ · Yuanjian Xu^1+^· Ruijuan Qiu^1^ · Jiangrui Chen^1^ · Linfeng Liu^1^ · Qijun Qian^1, 2, 3, 4*^**

^1^ Shanghai Cell Therapy Group Co., Ltd, 1535 Yuanguo Road, Shanghai 201805, Shanghai, China

^2^ Shanghai Cell Therapy Research Institute, 1585 Yuanguo Road, Shanghai 201805, Shanghai, China

^3^ Shanghai University Mengchao Cancer Hospital, 118 Qianyang Road, Shanghai 201805, Shanghai, China

^4^ School of Medicine, Shanghai University, 99 Shangda Road, Shanghai 200444, Shanghai, China

* Corresponding author: Zenghui Xu, E-mail: zenghuixu@163.com. Qijun Qian, E-mail: [qianqj@shcell.com](mailto:qianqj@shcell.com)

^+^ These authors contributed equally to this work


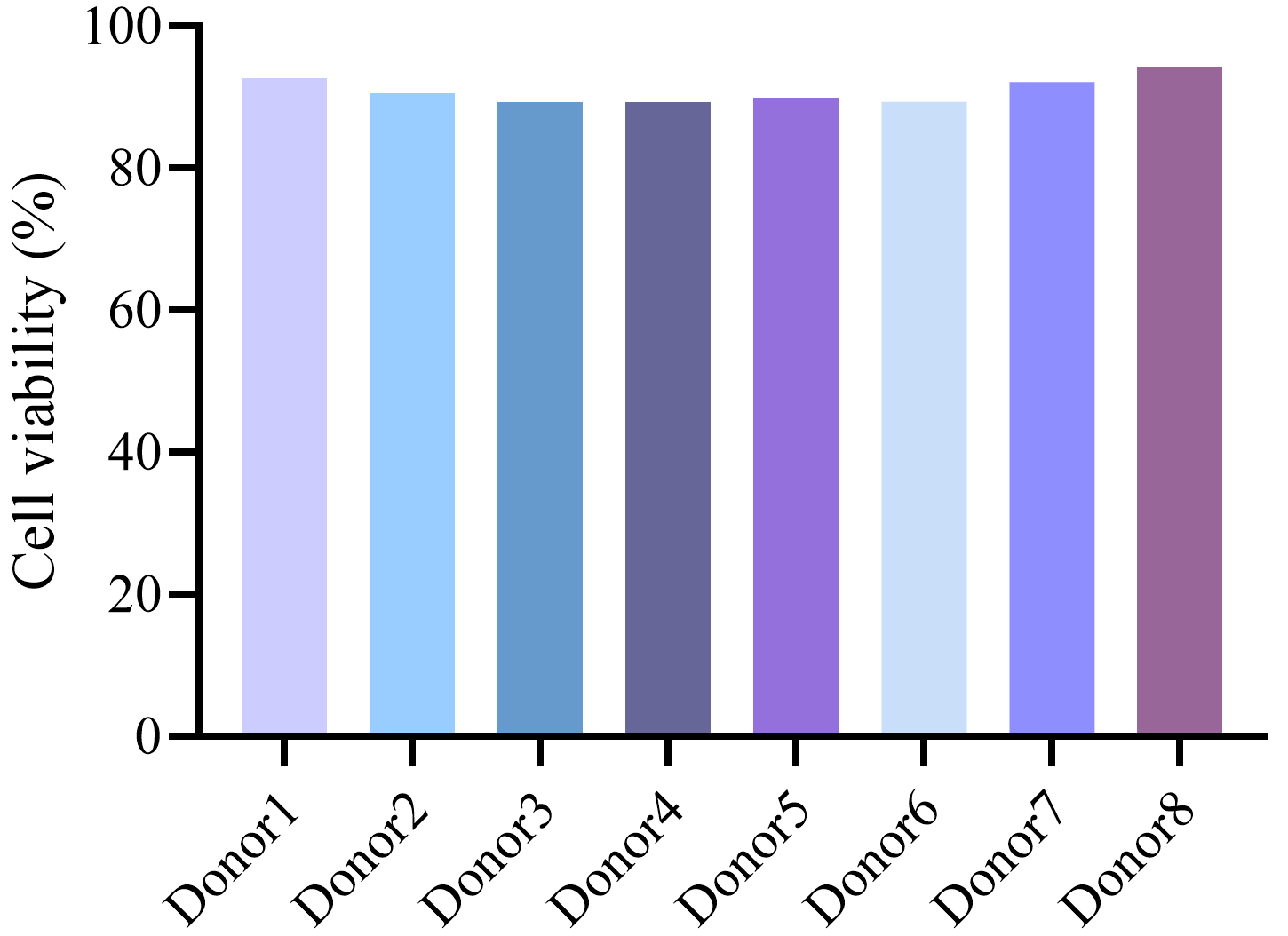


**Supplementary Figure 1：Cell viability of PBMCs after 3.5 years of cryopreservation**

PBMCs were separated via Ficoll density gradient centrifugation from peripheral blood. The collected PBMCs were added to cell cryopreservation media at 1~2 × 10^7^ cells/mL and stored in liquid nitrogen for 3.5 years. Cell viability was determined by AO/PI staining solution.

| **Group** | **1** | **2** |
| --- | --- | --- |
| Plasmid | MSLN+PD-1 Tiniplasmid, mRNA | MSLN Tiniplasmid, PD-1 Ti Tiniplasmid, mRNA |
| Cryopreservation duration | 35 days | 35 days |
| PBMCs | 3.8×10^7^ cells | 3.8×10^7^ cells |
| CAR-T cells viability (Day11) | 88.4% | 95.0% |
| Amplification (Day11) | 275.8 times | 470.7 times |
| CD3^+^ | 99.4% | 99.9% |
| CD3^+^CD4^+^ | 24.6% | 49.7% |
| CD3^+^CD8^+^ | 73.1% | 48.2% |
| CD3^+^CAR^+^ | 39.2% | 37.6% |

**Supplementary Table 1：Production of mesoCAR-T using different Tiniplasmids through electroporation**

PBMCs were separated via Ficoll from Leukapheresis. The collected PBMCs were added to cell cryopreservation media at 1~2 × 10^7^ cells/mL and stored in liquid nitrogen for 35 days. T Cell TransAct™ was used to activate T cells. Forty-eight hours later, activated T cells were electroporated using Tiniplasmid and mRNA, and then cultured for 11 days to generate mesoCAR-T. Tiniplasmid, a small backbone plasmid vector utilizing the toxin-antitoxin system as a marker for clonal screening.
